# Late Diagnosis of Maturity-Onset Diabetes of the Young (MODY) 12 With Catastrophic Consequences

**DOI:** 10.7759/cureus.13145

**Published:** 2021-02-05

**Authors:** Nuno R Carreira, Catarina Gonçalves, Alexandra Wahnon, Sara Dâmaso, Joao Martins

**Affiliations:** 1 Internal Medicine, Serviço de Medicina 2, Hospital de Santa Maria, Centro Hospitalar Universitário Lisboa Norte, Lisboa, PRT; 2 Oncology, Serviço de Medicina 2, Hospital de Santa Maria, Centro Hospitalar Universitário Lisboa Norte, Lisboa, PRT; 3 Internal Medicine: Diabetes and Endocrinology, Hospital de Santa Maria, Centro Hospitalar Universitário Lisboa Norte, Lisboa, PRT

**Keywords:** diabetes mody, chronic pancreatitis, pancreatic tumor, pancreatic adenocarcinoma, cancer, gene expression, atp binding cassette subfamily c member 8

## Abstract

Maturity-onset diabetes of the young (MODY) is a genetically and clinically heterogeneous group of diseases characterized by autosomal dominant monogenic non-ketogenic diabetes mellitus, usually with early-onset, with a prevalence of 1-5% of all diabetes cases.

A 72-year-old female was admitted with intestinal occlusion, anorexia, vomiting, and weight loss for four months. Medical history of type 2 diabetes mellitus, chronic pancreatitis with abnormal pancreatic development, and acute obstructive jaundice due to a mass in the head of the pancreas with duodenum extension four months before. Assuming surgically unresectable pancreatic neoplasm, digestive bypass surgery was performed. The pathologic examination of surgical specimens was negative for neoplasm.

Abdominal imaging showed the pancreatic mass, proximal bowel distension and ascites, which was negative for neoplastic cells. A percutaneous biopsy of the mass revealed adenocarcinoma. Palliative chemotherapy was started. Next-generation sequencing revealed the variant c.-8G>T in the 5’ untranslated region (UTR) region of the adenosine triphosphate (ATP) binding cassette subfamily C member 8 (ABCC8) gene in heterozygosity, associated with the MODY 12 subtype.

We report a possible case of MODY 12 diabetes with a phenotype not previously described: a non-neoplastic pancreatic mass that appears in a previously abnormally developed pancreas, with evolution to neoplasm along with the late development of diabetes mellitus. Although this ABCC8 gene mutation could be incidental, there could be a relationship between this mutation, pancreatic malformation, chronic pancreatitis and pancreatic neoplasm. Investigation of new phenotypes is critical, including the potential role of the ABCC8 gene in oncogenesis.

## Introduction

Diabetes mellitus (DM) is a common medical condition with a prevalence of 7.2-11.4% in individuals aged 20-79 years in 2015. Most patients present either type 1 auto-immune β-cell destruction (7-12% of all cases) or type 2 β-cell failure and insulin resistance forms (87-91% of all cases) [1]. Other subtypes of this disease are rare with the exception of gestational DM, occurring in about 17% of pregnant women [2].

Maturity-onset diabetes of the young (MODY) is a group of 13 monogenic forms of diabetes characterized by a primary defect in pancreatic β-cell function. It may account for 1-2% of all diabetes cases in Europe [3,4]. Mutations in genes responsible for the development and function result in cellular dysfunction and are typically transmitted in an autosomal dominant pattern. Nonetheless, recessive transmission with homozygosity is possible in MODY 2, 4 and 6 subtypes resulting in a more serious phenotype. [5] Specifically, adenosine triphosphate (ATP) binding cassette subfamily C member 8 (ABCC8) gene mutations are associated with MODY 12.

Clinically this disease has an early onset, usually before 25 years old, with some forms emerging during the neonatal period (MODY 9-13) [5,6]. Patients may present mild asymptomatic hyperglycemia from childhood to adulthood, with progressive development to clinical DM. Clinical features such as obesity and ketoacidosis are rare. First-line treatment relies on oral hypoglycemic agents or insulin according to MODY subtype [5].

Besides impaired function of pancreatic β-cells, MODY is also associated with agenesis, hypoplasia or abnormal development of the pancreas (MODY 4, 5 and 6), exocrine pancreatic dysfunction (MODY 5 and 8), pancreatic malignancy (MODY 7), renal abnormalities/failure at young age and genital abnormalities (MODY 5). MODY 9-13 are associated with neonatal diabetes [5,6]. The role of several mutations is still poorly understood, which may unveil new and unknown clinical features.

## Case presentation

A 72-year-old Caucasian female was assisted at an endocrinology appointment in the context of presumably long-standing type 2 DM. She was first diagnosed by her general practitioner through a routine medical workup by the age of 60, and glycemic control was achieved with diet, exercise and an oral secretagogue agent (gliclazide 30 mg twice daily). Hemoglobin A1c (HbA1c) was maintained around 7% and no macro or microvascular complications were reported.

Growth and pubertal development were uneventful, with no relevant gynecological events and no previous pregnancies. The patient reported never being overweight. Since childhood, she suffered from unspecified abdominal complaints and developed steatorrhea in early adulthood. Imaging studies identified “pancreatic calcifications” and “abnormal pancreatic development”, not otherwise specified, and a diagnosis of chronic pancreatitis was established. Pancreatic enzymes and dietary modifications were prescribed, providing symptom remission. The patient had no other diseases and no family history of diabetes.

At presentation, the patient was 1.50 m in height, underweight (BMI 16.8 kg/m2), with a normal physical examination. Regarding this clinical course, notably the development of diabetes in a patient that had always had good metabolic control with weak secretagogues and diet, with no evidence of metabolic syndrome, negative glutamic acid decarboxylase autoantibodies (GAD antibodies), and abnormal pancreatic development, the diagnosis of MODY was suspected. Genetic testing for MODY was then requested.

During follow-up, the patient presented an acute abdominal crisis with diffuse pain, acute intestinal obstruction and jaundice, which required admission to the emergency room. Laboratory study revealed cytocholestatic pattern. An abdominal ultrasound revealed a large heterogeneous and calcified mass (34x22 mm) in the head of the pancreas causing choledochus obstruction, and intraperitoneal fluid. There were no other reported abnormalities, including the liver, with no evidence of ascites or adenopathies. After a presumptive diagnosis of unresectable pancreatic cancer with bowel occlusion, she underwent derivative surgery with construction of a Billroth type 1 anastomosis and partial resection of the pancreatic mass for pathological analysis. No neoplastic tissue was found in the pancreatic mass, abdominal ganglia, gastric and duodenal mucosa. Serum tumor markers including carcinoembryonic antigen (CEA) and cancer antigen (CA) 19-9 were normal. The patient was discharged asymptomatic.

**Figure 1:**
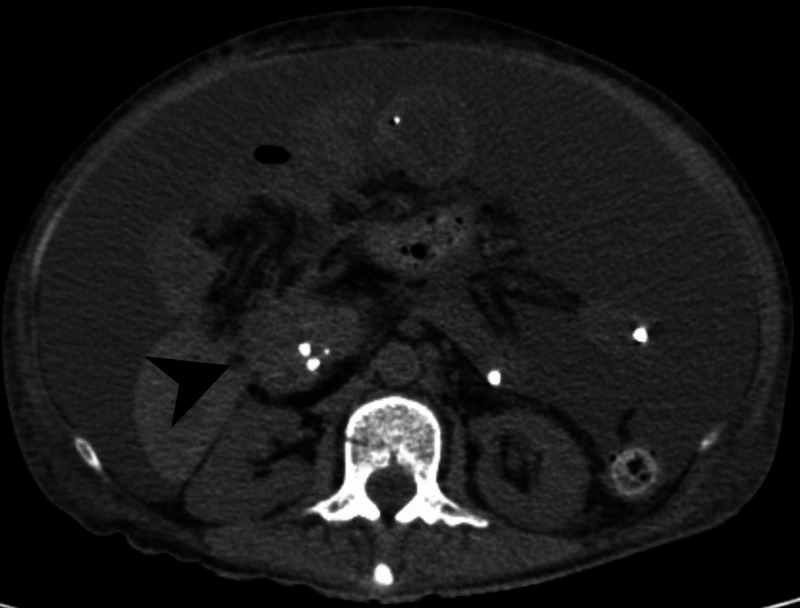
Abdominal Computed Tomography Black arrow highlights cephalic pancreatic mass

Four months later, the patient was again admitted to the emergency room with severe anorexia, vomiting and weight loss >20% of body weight. Physical examination showed signs of dehydration and cachexia, and a tender and painful abdomen. To investigate a possible intestinal occlusion an abdominal tomography was performed (Figure 1), which showed the remaining mass in the cephalic portion of the pancreas, moderate ascites, extensive diffuse lesions suggestive of peritoneal carcinomatosis and gastric, jejunal and proximal ileal distension with a transition point on the left flank. In the absence of surgical indication, medical therapy was instituted, without resolving the obstruction.

There was an increase in intraperitoneal fluid, without neoplastic cells found on ascitic fluid examination. CA 19-9 was then elevated (271 U/mL - reference range: 0-37U/mL). Fine needle aspiration biopsy of the remaining pancreatic mass allowed this time a diagnosis of pancreatic adenocarcinoma. Palliative chemotherapy was started, however the patient died after one course due to disease progression and degradation of general status.

Later on, results of multiple genetic screening for MODY revealed the presence of the c-8G>T variant in the ABCC8 gene in heterozygosity. ABCC8 gene mutations are associated with MODY 12.

## Discussion

Several questions arise from this case report. Initially, this patient presented DM that did not fit into type 1 or type 2 classical features: the patient had always been normal or underweight, with no family history of DM; good metabolic control was maintained with diet, exercise and weak secretagogue drugs; GAD antibodies were absent, making unlikely the diagnosis of Latent Autoimmune Diabetes of the Adult (LADA) [1,2,7], and no other syndromic or endocrine disease was present [7,8].

Table 1 describes MODY clinical features.

**Table 1:** Clinical features of maturity onset diabetes (MODY) of the young (adapted from [6])

Table 1
One of the following:
• Diabetes diagnosed ≤ 30 years.
• Diabetes diagnosed ≤ 45 years in people without obesity/ insulin resistance/ metabolic syndrome.
• Diabetes diagnosed ≤ 45 years and a family history of diabetes in ≥ 2 generations in an autosomal dominant fashion.
And in absence of:
• Diabetic ketoacidosis.
• Pancreatic islet autoantibodies.
• No endogenous insulin production outside the honeymoon period of about 3 years (e.g. undetectable C-peptide).
Additionally, specific features of MODY subtypes may justify genetic testing:
• Glycosuria at blood glucose levels < 10 mmol/l.
• Marked sensitivity to sulfonylurea derivatives.
• Diabetes associated with extra pancreatic features: non-diabetic renal disease, renal anomalies, genital anomalies, abnormal liver function tests.

The patient presented evidence of abnormal pancreatic development since early life, diagnosed as chronic pancreatitis. An insufficient exocrine pancreatic function could justify the short stature due to malabsorption [3].

The obvious diagnosis would be DM secondary to long-standing chronic pancreatitis, with decreased insulin reserve. However, the clinical evidence for chronic pancreatitis was mild since the patient maintained good health and nutrition status with a basic dietary plan and pancreatic enzymes. Development of DM in cases of chronic pancreatitis, also known as type 3c DM, is mainly caused by destruction of islet cells by pancreatic inflammation. Severe exocrine pancreatic insufficiency is usually evident at that time due to pancreatic tissue destruction [9]. 

MODY could be a possible alternative diagnosis. Patients with MODY are often misdiagnosed as type 1 or type 2 diabetics. An increasing number of MODY subtypes are now recognized, although the underlying pathogenesis is poorly understood. Some mutations are recognized to impair β-cell uptake of blood glucose, disturbing cell metabolism while others occur in homeobox genes critical for pancreatic development. The pathological role of other gene mutations remains unknown [4,5].

This patient presented a mild long-standing DM managed with minimal doses of weak secretagogue agents which fits the diagnosis of either DM secondary to chronic pancreatitis or a MODY.

Two main reasons could be argued against the MODY diagnosis: the initial diagnosis later in life and absence of family history of DM. Nonetheless, new mutations can occur in individuals without family history of MODY. In favor of this diagnosis there is the strong fact that abnormal pancreatic development was present, which would be a strong reason for diabetes mellitus, particularly MODY types 5-8 [5].

In our patient multiple gene screening for MODY was performed. All 15 genes were analyzed through next-generation sequencing (NGS) - ABCC8, APPL1, BLK, CEL, GCK, HNF1A, HNF1B, HNF4A, INS, KCNJ11, KLF11, NEUROD1, PAX4, PDX1 and SCL16A1 - and a variant c.-8G>T in the 5’UTR region of the ABCC8 gene in heterozygosity (NM_000352.6) was identified [10]. This is a variant of unknown significance, although it has been reported as probably benign. It is a relatively rare variant in the healthy population (minor allele frequency (MAF); gnomAD: 0.25%) and most in silico predictions suggest pathogenicity [11]. ABCC8 mutations have been associated with MODY 12, autosomal recessive (mostly) or dominant (MIM 256450) transmission [12], with transient or permanent mild diabetes mellitus partially responding to secretagogues [3,6].

The ABCC8 gene is located in chromosome 11 (11p15.1) and encodes the adenosine triphosphate (ATP) binding cassette subfamily C member 8, a regulatory subunit of the ATP-dependent K+ channel, that is the potential sulfonylurea receptor [7]. Inactivating mutations have been associated with neonatal DM while activating mutations may result in serious neonatal hypoglycemia [10].

ABCC8 mutations have also been associated with oncogenesis [13]. Pancreatic ductal adenocarcinoma (PDAC) is the fourth most frequent cause of cancer-related deaths worldwide with a five-year overall survival <8% [14]. The majority of pancreatic carcinomas are sporadic and risk factors include age, tobacco, alcohol consumption, obesity, chronic pancreatitis and DM [15]. Patients with chronic pancreatitis present an odds ratio of 2.7 for PDAC, although the causal association between both entities is hard to establish since pancreatitis in these patients may represent an initial manifestation of PDAC, as opposed to a contributing cause [14]. Some meta-analyses have demonstrated associations between DM and PDAC, with an odds ratio of approximately 2. Similar to pancreatitis, a causal link has not yet been established. DM can emerge as a manifestation of PDAC, blurring its true role as a risk factor [14,15]. Sixty to 70% of pancreatic cancer arises in the head of the pancreas. If the tumor grows locally into the duodenum can result in gastrointestinal obstruction [15].

A small portion of PDAC is caused by familial syndromes with germline mutations. Increased risk for PDAC is linked to mutations in BRCA2, ATM, PALB2, STK11 and DNA mismatch repair genes. The majority of patients however develop sporadic PDAC through accumulation of somatic mutations. Mutations in ABCC8 have not been described in the literature as drivers for PDAC, although a recent analysis found an association of this gene with the development and prognosis of pancreatic neuroendocrine tumors [16]. Additionally, it was previously suggested that upregulation and downregulation of different ABC family genes in PDAC may have a role in tumor progression and treatment resistance [13].

This patient had both chronic pancreatitis and MODY 12 with an abnormal pancreatic development as risk factors for pancreatic cancer. The first approach to the pancreatic mass showed no evidence of malignancy in the surgical specimens and no evidence of other sites of dissemination on abdominal tomography. Clinical deterioration months later required new diagnostic procedures. A new abdominal tomography revealing multiple implants suggestive of peritoneal carcinomatosis and elevated serum CA 19-9 brought again the hypothesis of an advanced pancreatic carcinoma, which was then confirmed by fine-needle aspiration biopsy.

It should be noted that the patient’s pancreatic mass was certainly long-standing as suggested by its heterogeneous nature and calcifications, and part of it probably did not correspond to neoplasm as suggested by initial negative pathological analysis. Pancreatic adenocarcinoma eventually progressed with extensive peritoneal carcinomatosis and intestinal occlusion and finally death.

Although the patient's global presentation was more typical of MODY 5-8 subtypes, this mutation on the ABCC8 gene, recognized in MODY 12, may have clinical and pathological significance, previously unknown upon scientific knowledge.

This patient gene screening results do not explain or exclude this diagnosis, and further studies are needed in order to clarify the clinical significance of the variant found, particularly studies on family segregation. However, we speculate that this patient had a rare variation in the ABCC8 gene that caused pancreatic maldevelopment, chronic pancreatitis, MODY, and an increased risk for pancreatic adenocarcinoma, ending with catastrophic results.

## Conclusions

MODY should be considered as a differential diagnosis in the investigation of patients with diabetes and pancreatic mass, since this disease may lead to pancreatic tumor, which could have catastrophic consequences. More studies are needed to identify new potential genes associated with MODY, and pathogenic link of new discovered ones should be investigated. Besides, the potential role of ABCC8 gene mutations in oncogenesis must be investigated. Finally, the authors highlight the relevance of genetic testing even in patients without history of diabetes, not only for the early recognition of MODY subtypes but also for establishing successful treatments and family counseling.
